# Baseline Ga-68 PSMA PET-Derived Primary Tumor Parameters in Patients with Prostate Cancer and Their Association with Clinical Risk Stratification and Clinicopathologic Features

**DOI:** 10.1055/s-0044-1787733

**Published:** 2024-06-14

**Authors:** Özge Vural Topuz, Ayşegül Aksu

**Affiliations:** 1 Department of Nuclear Medicine, University of Health Sciences, Başakşehir Cam and Sakura City Hospital, Istanbul, Türkiye; 2 Department of Nuclear Medicine, Atatürk Training and Research Hospital, İzmir Kâtip Çelebi University, Izmir, Türkiye

**Keywords:** prostate cancer, ^68^ Ga-PSMA PET/CT, Gleason score, D'Amico risk class, Candiolo nomogram

## Abstract

**Aim** This article evaluates whether parameters derived from the gallium-68-labeled prostate-specific membrane antigen ( ^68^ Ga-PSMA) positron emission tomography/computed tomography (PET/CT) imaging studies of primary prostate cancer (PCa) lesions were associated with Gleason score (GS), D'Amico risk class, Candiolo nomograms, and the metastatic status of the disease.

**Methods** We retrospectively evaluated newly diagnosed PCa patients who underwent ^68^ Ga-PSMA PET/CT before therapy. Age, baseline serum prostate-specific antigen (PSA), and metastatic status were recorded. Maximal standardized uptake value (SUVmax), mean SUV (SUVmean), total lesion PSMA (TL-PSMA), and PSMA-derived tumor volume (PSMA-TV) were analyzed. The patients were grouped according to GS (GS ≤ 7 and GS ≥ 8), D'Amico risk classes (low intermediate and high-risk), and also based on their results with the Candiolo nomogram which normally creates five risk classes. For Candiolo classes, very-low risk and low-risk patients were pooled into the low-risk Candiolo (LRC) group, high and very high-risk patients were pooled into the high-risk Candiolo (HRC) group. The intermediate-risk Candiolo group was utilized as-is (IRC).

**Results** Mean age was 67 ± 8 years, median PSA value was 14.3 (3–211). There were 82 patients with GS ≤ 7 and 38 patients with GS ≥ 8; intermediate D'Amico class comprised 32 patients, while the high D'Amico class comprised 88 patients. For Candiolo, there were 23 LRC, 40 IRC, and 57 HRC patients. PSMA-positive metastases were detected in 44 (36.7%) patients. The SUVmean, SUVmax, PSMA-TV, and TL-PSMA values of the primary tumor demonstrated significant differences when compared according to classifications for GS, D'Amico, LRC versus HRC, and metastatic versus nonmetastatic patients. Of note, TL-PSMA was the only parameter that varied significantly among all risk groups.

**Conclusion** Primary tumor parameters obtained from baseline ^68^ Ga-PSMA PET/CT are useful to distinguish PCa patients in terms of GS, D'Amico, Candiolo nomogram, and metastatic states. TL-PSMA appears to be the best parameter as it is the only parameter that can distinguish all risk groups from each other.

## Introduction

Prostate cancer (PCa) is one of the most common cancers in men worldwide and continues to be an important cause of death in many regions. 1 Risk stratification among patients with PCa is crucial since it is a heterogeneous disease ranging from prostate-localized disease to castration-resistant PCa, and especially because the different stages of the disease directly affect management and prognosis. In traditional PCa risk classification, patients are classically divided into risk groups according to the D'Amico risk classes which categorize patients into three groups based on pretreatment prostate-specific antigen (PSA) level, clinical stage, and biopsy Gleason score (GS). 2

A new classification tool, the “Candiolo nomogram,” was published in 2016. This nomogram has been suggested to be superior to the D'Amico approach in predicting PCa recurrence after radiotherapy (RT). It leverages five easily accessible pretreatment parameters as clinical predictors, including age, PSA, clinical-radiological stage (cT stage), biopsy GS, and percentage of biopsy positive cores (%PC). 3

Prostate-specific membrane antigen (PSMA) is a transmembrane protein with significantly increased expression in PCa cells or metastasis. 4 In recent years, positron emission tomography/computed tomography (PET/CT) with gallium-68-labeled PSMA ( ^68^ Ga-PSMA) has become the standard evaluation method routinely used to detect PCa and determine stage, response to treatment, and biochemical recurrence. 5 Additionally, maximum standardized uptake value (SUVmax) obtained from ^68^ Ga-PSMA PET/CT is a semiquantitative parameter that has gained widespread use for tumor evaluations. Recent studies have suggested that volume-based parameters obtained via ^68^ Ga-PSMA PET/CT, such as PSMA-derived tumor volume (PSMA-TV) and total lesion PSMA (TL-PSMA), can reflect the tumor burden of PCa patients, and thus, may provide more accurate results in the evaluation of prognosis and monitoring of treatment response. 6

The aim of this study was to investigate the potential relationships between baseline ^68^ Ga-PSMA PET/CT-derived primary tumor features and classical characteristics such as GS, other pretreatment risk stratifications, and metastatic status among patients with PCa.

## Material and Methods

### Study Setting, Design, and Patient Selection

We retrospectively reviewed PCa patients who had pretreatment ^68^ Ga-PSMA PET/CT imaging in our clinic between January 2021 and January 2022. Inclusion criteria were: confirmation of prostate adenocarcinoma by pathological examination, having undergone baseline ^68^ Ga-PSMA PET/CT for staging, availability of clinical history, age, GS, and serum PSA levels within 2 weeks before imaging. Exclusion criteria were: having a pathological diagnosis other than prostate adenocarcinoma, having received any previous treatment, and absence of data concerning GS, histological results, or baseline PSA values. Serum PSA levels (ng/mL), cT tumor grading (based on biopsy results), and %PC were recorded in all participants. All procedures performed in this retrospective study were in accordance with the ethical standards of the 1964 Helsinki Declaration or comparable ethical standards. Written informed consents were obtained from all patients.

### Risk Stratification Systems

Gleason patterns were grouped according to the Gleason Grading System suggested by the 2014 International Society of Urological Pathology consensus conference. 7 According to GS, the patients were grouped as patients with GS ≤ 7 and patients with GS ≥ 8.

For D'Amico risk classes, the patients were divided into three categories based on pretreatment PSA, clinical stage, and biopsy GS results: low risk (PSA < 10 ng/mL and cT1–cT2a and a biopsy GS ≤ 6), intermediate risk (PSA 10–20 ng/mL or cT2b or biopsy GS 7), and high risk (PSA > 20 ng/mL or clinical stage ≥ cT2c or biopsy GS ≥ 8). 2

According to the Candiolo nomogram, the five parameters were categorized as follows: age ≥ 70 years or age < 70 years; PSA < 7 ng/mL, 7 to 15 ng/mL, or > 15 ng/mL; clinical-radiological T stage cT1, cT2, or cT3-cT4; biopsy GS ≤ 6, 3 + 4, 4 + 3, 8, or 9 to 10; %PC 1 to 20%, 21 to 50%, 51 to 80%, or 81 to 100%. The %PC was calculated by multiplying 100 by the number of positive cores containing PCa (of any length) and dividing by the total number of cores sampled. The patients were divided into five risk classes: very low-risk, low-risk, intermediate-risk (intermediate-risk Candiolo) (IRC), high-risk, and with very high-risk. 3 Very low-risk and low-risk patients were combined to create the low-risk Candiolo (LRC) group, while high-risk and very high-risk patients were combined to create the high-risk Candiolo (HRC) group. This approach was deemed necessary due to the small size of the combined groups.

Patients were grouped as metastatic and nonmetastatic according to their metastatic status.

### ^68^ Ga-PSMA Imaging Procedure

The ^68^ Ga-PSMA used for imaging was synthesized by employing a radiopharmaceutical practice module (Scintomics, Germany) equipped with a fully automated synthesis unit. After production, labeling efficacy was assessed with high-performance liquid chromatography. Imaging procedures are briefly described as follows: 1 hour after the administration of an average of 3.1 mCi (115 MBq) of ^68^ Ga-PSMA, the patients underwent noncontrast CT imaging (0.5 mm slice thickness) (Philips Gemini TF PET/CT, Eindhoven, The Netherlands). After CT, whole-body PET images of 10 to 12 bed positions were obtained with a 1.5-minute emission per bed position from vertex to feet.

### Image Analysis and Calculation of Volumetric Parameters

All lesions that were considered malignant by two experienced nuclear medicine specialists and showed PSMA expression higher than background activity (except for areas of physiological involvement and benign lesions) were considered positive. Semiautomatic volumes of interest (VOIs) were taken from the primary lesion using a 40% SUV threshold within the lesion image area in all three planes. The SUVmax of the primary tumor refers to the highest ^68^ Ga-PSMA uptake in a VOI, while SUVmean refers to the average SUV concentration. Similar to metabolic tumor volume (MTV), PSMA-TV defines the tumor volume that demonstrates PSMA uptake greater than a threshold of 40% of SUVmax in the VOI.

SUVmax, SUVmean, and MTV results were automatically generated from VOIs by the workstation. For each lesion, TL-PSMA was calculated by multiplying the corresponding SUVmean and PSMA-TV values.

### Statistical Analysis

For statistical analysis, the Statistical Package for the Social Sciences (SPSS) v. 22.0 was used. A value of *p* < 0.05 was considered significant. Normally distributed data were summarized as mean ± standard deviation, and nonnormally distributed data were summarized as median (range). Differences between continuous variables among the compared groups were evaluated with the Mann–Whitney *U* test, and categorical variables were evaluated with appropriate chi-square tests or the Fisher's exact test. Directional relationships between continuous parameters were analyzed by calculating the Spearman's correlation coefficient. Analyses involving > two groups were performed by employing one-way analysis of variance or the Kruskal–Wallis *H* test. When a significant difference was found with the overall comparison with these tests, post hoc pairwise comparisons were performed to identify the groups causing the significant difference. To determine the tests to be used for post hoc correction, we performed the Levene test and determined homogeneity of variances. If the variances were homogeneous, the Bonferroni correction was used in pairwise comparisons, whereas the Tamhane T2 test was used when variances were not homogenous. Logistic regression was used to perform multivariable analysis; parameters that correlated with each other were not included in the model.

## Results

A total of 120 treatment-naive biopsy-proven PCa patients with a mean age of 67 ± 8 years (46–87) were included in the study. Median PSA value was 14.3 (3.0–211.0). GS values were 3 + 4 in 45 (37.5%), 4 + 3 in 37 (30.8%), 4 + 4 in 20 (16.7%), 4 + 5 in 15 (12.5%), 5 + 4 in 2 (1.7%), and 5 + 5 in 1 (0.8%) patients. Clinicopathologic characteristics of the study population are presented in Table 1 .

**Table 1:** Clinicopathologic characteristics of study population

Variables	Result
Age (y), mean ± SD	67 ± 8 (46–87)
PSA, median (range)	14.3 (3–211)
Gleason score	
3 + 4	45 (37.5%)
4 + 3	37 (30.8%)
8	20 (16.7%)
4 + 5	15 (15%)
5 + 4	2 (1.7%)
5 + 5	1 (0.8%)
cT stage	
T2	53 (44.2%)
T3	58 (48.3%)
T4	9 (7.5%)
PNI	77 (64.2%)
LVI	10 (8.3%)
Any PSMA-positive metastases	44 (36.7%)
Candiolo risk class	
Very-low	2 (1.7%)
Low	21 (17.5%)
Intermediate	40 (33.3%)
High	28 (23.3%)
Very-high	29 (24.2%)
D'Amico risk class	
Intermediate	32 (27.7%)
High	88 (73.3%)

Abbreviations: cT stage, clinical-radiological stage; LVI, lymphovascular invasion; PNI, perineural invasion; PSA, prostate-specific antigen; PSMA, prostate-specific membrane antigen; SD, standard deviation.

There were 82 patients categorized as GS ≤ 7 and 38 patients as GS ≥ 8. According to the D'Amico risk classification, 88 (73.3%) patients were in the high-risk group and 32 patients were in the intermediate-risk group.

In accordance with the Candiolo nomogram classification, 2 (1.7%) patients were defined as very low-risk, 21 (17.5%) as low-risk, 40 (33.3%) as intermediate-risk, 28 (23.3%) as high-risk, and 29 as very high-risk (Candiolo nomogram scores summarized in Table 2 ). After pooling, there were 23 patients in the LRC group, 40 patients in the IRC group, and 57 patients in the HRC group.

**Table 2:** Candiolo nomogram scores based on other parameters

**bGS**	**≤6**	**3** **+** **4**	**4** **+** **3**	**8**	**9–10**
Points	0	35	48	76	106
**cT**	**cT1**	**cT2**	**cT3–4**		
Points	0	17	58		
**PSA**	**<7**	**7–15**	**>15**		
Points	0	42	96		
**%PC**	**1–20%**	**21–50%**	**51–80%**	**81–100%**	
Points	0	29	50	81	
**Age**	**≥70 y**	**<70 y**			
Points	0	22			
**Risk-class**	**Very-low**	**Low**	**Intermediate**	**High**	**Very-high**
Total points	0–56	57–116	117–193	194–262	263–363

Abbreviations: bGS, biopsy Gleason score; cT stage, clinical-radiological stage; PSA, prostate-specific antigen; %PC, percentage of biopsy positive cores.

PSMA-positive metastases were detected in 44 of the 120 (36.7%) patients. Seventeen patients (14.2%) had only pelvic lymph node metastases. Skeletal metastases were shown in 27 (22.5%) patients and 15 of these patients also had pelvic lymph node metastases.

The SUVmean values of the primary tumor demonstrated significant differences between GS subgroups (GS ≤ 7 vs. GS ≥ 8), between D'Amico subgroups (intermediate vs. high-risk), between the LRC and HRC subgroups of the Candiolo nomogram, and between the metastatic and nonmetastatic groups ( *p* < 0.05 for all) ( Table 3 ).

**Table 3:** SUVmean values according to clinicopathological features

Variables		SUVmean	*p*
Gleason score	≤ 7	5.2 (1.8–22)	0.004
	≥ 8	8.5 (1.6–42.3)	
D'Amico risk	Intermediate	3.9 (2–17.4)	0.001
	High	7.8 (1.6–42.3)	
Candiolo nomogram	LRC	5.1 ± 3.8	LRC versus HRC *p* < 0.001
	IRC	6.3 ± 4.3	
	HRC	10.4 ± 7.1	
Metastases	Nonmetastatic	5.1 (1.8–22)	0.001
	Metastatic	9.2 (1.6–42.3)	

Abbreviations: HRC, high-risk Candiolo; IRC, intermediate-risk Candiolo; LRC, low-risk Candiolo; SUVmean, mean standardized uptake value.

The SUVmax values of the primary tumor were found to be significantly different for comparisons within the GS and D'Amico subgroups, as well as between the LRC and HRC groups of the Candiolo nomogram, and between metastatic and nonmetastatic groups ( *p* < 0.05 for all) ( Table 4 ).

**Table 4:** SUVmax values according to clinicopathological features

Variables		SUVmax	*p*
Gleason score	≤ 7	8.7 (2.6–35)	0.003
	≥ 8	15.5 (2.6–81.4)	
D'Amico risk	Intermediate	6.2 (3.3–27)	0.001
	High	13.5 (2.6–81.4)	
Candiolo nomogram	LRC	8.2 ± 6.1	LRC versus HRC *p* < 0.001
	IRC	10.8 ± 7.3	
	HRC	17.9 ± 12.7	
Metastases	Nonmetastatic	8.5 (2.6–35)	< 0.001
	Metastatic	16.1 (2.6–81.4)	

Abbreviations: HRC, high-risk Candiolo; IRC, intermediate-risk Candiolo; LRC, low-risk Candiolo; SUVmax, maximal standardized uptake value.

The PSMA-TV values obtained for the primary tumor were also revealed to be significantly different between GS subgroups, D'Amico subgroups, in the LRC versus HRC comparison, and in the metastatic versus nonmetastatic comparison ( *p* < 0.05 for all) ( Table 5 ).

**Table 5:** PSMA-TV values according to clinicopathological features

Variables		PSMA-TV	*p*
Gleason score	≤ 7	6.2 (0.3–33.5)	< 0.001
	≥ 8	12.9 (1.5–352)	
D'Amico risk	Intermediate	4 (1.6–16.7)	< 0.001
	High	9.6 (0.3–352)	
Candiolo nomogram	LRC	5.2 ± 3.5	LRC versus HRC *p* < 0.005
	IRC	7.2 ± 5.1	
	HRC	22.3 ± 48	
Metastases	Nonmetastatic	6.3 (0.3–33.5)	0.001
	Metastatic	10.5 (1.9–352)	

Abbreviations: HRC, high-risk Candiolo; IRC, intermediate-risk Candiolo; LRC, low-risk Candiolo; PSMA **-** TV, prostate-specific membrane antigen-derived tumor volume.

**Fig. 1:**
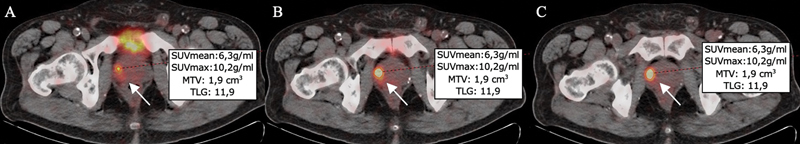
Pretreatment gallium-68-labeled prostate-specific membrane antigen (Ga-68-PSMA) positron emission tomography/computed tomography (PET/CT) images of a 68-year-old prostate cancer (PCa) patient categorized as Gleason score (GS): 4 + 3, D'Amico intermediate risk, low-risk Candiolo (LRC), nonmetastatic tumor. Summary of maximal standardized uptake value (SUVmax), mean SUV (SUVmean), metabolic tumor volume (MTV), and total lesion glycolysis (TLG) values derived from the primary prostate tumor (arrow) on axial fused pretreatment 68Ga-PSMA PET/CT images of a patient with low risk ( **A** – **C** ).

**Fig. 2:**
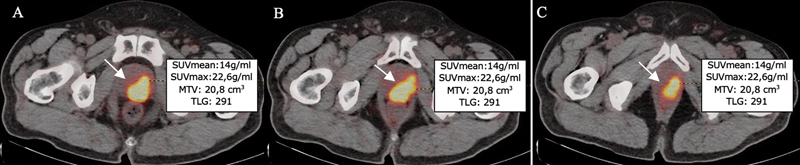
Pretreatment gallium-68-labeled prostate-specific membrane antigen (Ga-68-PSMA) positron emission tomography/computed tomography (PET/CT) images of a 59-year-old prostate cancer (PCa) patient categorized as Gleason score (GS): 4 + 5, D'Amico high risk, high-risk Candiolo (HRC), metastatic tumor. Summary of maximal standardized uptake value (SUVmax), mean SUV (SUVmean), metabolic tumor volume (MTV), and total lesion glycolysis (TLG) values derived from primary prostate tumor (arrow) on axial fused pretreatment 68Ga-PSMA PET/CT images of a patient with low risk ( **A** – **C** ).

Finally, we also determined that the values for TL-PSMA calculated for the primary tumor were significantly different when compared within subgroups created based on GS, D'Amico, Candiolo, and metastatic status ( *p* < 0.05 for all) ( Fig. 1 and Fig. 2 ). Of note, TL-PSMA values demonstrated significant post hoc differences for all pairwise analyses between the three Candiolo subgroups. As such, TL-PSMA was the only parameter that showed significant differences between all risk subgroups ( Table 6 ).

**Table 6:** TL-PSMA values according to clinicopathological features

Variables		TL-PSMA	*p*
Gleason score	≤ 7	30.5 (0.8–380.4)	< 0.001
	≥ 8	97.5 (10.8–3168)	
D'Amico risk	Intermediate	19.8 (6.4–136.57)	< 0.001
	High	58.6(0.8–3168)	
Candiolo nomogram	LRC	22.8 ± 17.8	LRC versus IRC *p* : 0.032
	IRC	40.1 ± 34.1	HRC versus IRC *p* : 0.012
	HRC	227.1 ± 469.7	LRC versus HRC *p* : 0.005
Metastases	Nonmetastatic	32.3(0.8–367.8)	< 0.001
	Metastatic	103.7 (10.8–3168)	

Abbreviations: HRC, high-risk Candiolo; IRC, intermediate-risk Candiolo; LRC, low-risk Candiolo; TL-PSMA, total lesion prostate-specific membrane antigen.

## Discussion

In this study, we investigated the potential relationships between primary tumor parameters obtained from baseline ^68^ Ga-PSMA PET/CT imaging and classical risk-stratification outcomes such as GS, D'Amico, and Candiolo nomogram, as well as the metastatic status of PCa patients.

We were able to validate prior publications reporting that patients with GS > 7 show significantly higher PSMA uptake than those with GS 6 and 7. 8 9 10 These studies only investigated SUVmax and did not analyze quantitative PET parameters; however, in our study, all baseline ^Ga^ 68 PSMA PET/CT primary tumor-derived parameters, including quantitative parameters, were analyzed and significantly higher values were found in patients with GS ≥ 8 compared with those with a GS of ≤ 7.

Since the D'Amico risk classification remains as the main clinical assessment used to guide treatment decisions, there are many published studies in the literature investigating the relationship between D'Amico risk groups and the ^68^ Ga-PSMA PET/CT parameters of PCa patients. While some of these studies only evaluate SUV parameters, 9 10 11 there are several studies which have also included semiquantitative parameters—similar to our study. 12 13

Although various models have been proposed in recent years for early prediction of PCa course, most of these have gained very limited clinical use due to lack of external validation. Gabriele et al developed the Candiolo nomogram, which classifies PCa patients undergoing radical RT, which has been determined to be able to precisely predict the risk of biochemical recurrence. 14 In another study with thousands of PCa patients treated with external-beam RT, Gabriele et al classified patients into five different “Candiolo” risk classes and concluded that this classification appeared better at predicting PCa recurrence after RT—when compared with the D'Amico approach. 3 In another study comprising 561 PCa patients receiving therapeutic RT, Gabriele et al performed an external validation for the Candiolo nomogram to assess clinical utility before radical RT in PCa patients. 14 Utsumi et al concluded that reclassification of traditional high-risk PCa patients with the Candiolo nomogram increased the prediction performance of biochemical recurrence after carbon-ion RT and androgen deprivation therapy. 15 In our study, we report the relationship between this externally validated and increasingly relevant nomogram and features/parameters obtained from baseline ^68^ Ga-PSMA PET/CT. Because nomograms combine relevant prognostic variables, individual patient prediction may be more accurate relative to the risk groups created by traditional classification systems. It is not surprising that a prediction model with more variables will have higher prediction accuracy, but such a model with more unique variables may not be easy to use in clinical practice. That being said, it is evident that the Candiolo nomogram is not an overly complex model when compared with the National Comprehensive Cancer Network or D'Amico risk classes. 15 We have shown that all quantitative parameters obtained from PSMA PET are able to distinguish the HRC and LRC groups, and in addition, we have shown that TL-PSMA values can also distinguish the IRC group.

Similar to previous studies published in the literature, all baseline ^68^ Ga-PSMA PET/CT parameters obtained from the primary lesion were significantly higher in high-risk PCa patients. 8 Although SUVmax is the most commonly used semiquantitative parameter in PET/CT, recent studies investigating ^68^ Ga-PSMA PET/CT-derived volumetric parameters such as PSMA-TV and TL-PSMA showed that they can more accurately reflect tumor burden. Based on these findings and our results, it appears that these parameters can more accurately predict PCa-related risks and improve prognostication and the assessment of treatment response. 6 Our data showed that TL-PSMA was the only parameter that could distinguish IRC from other Candiolo risk groups (LRC and HRC). We believe that the superiority of TL-PSMA compared with PSMA-TV (which only accounts for MTV) is a direct result of the fact that TL-PSMA also considers the degree of PSMA expression in the tumor, and therefore, it facilitates the integration of the molecular profile of PCa in risk stratification.

In our study, all ^68^ Ga-PSMA PET/CT parameters were significantly higher in the metastatic groups than in the nonmetastatic groups. In the study of Liu et al, SUVmax, PSMA-TV, and TL-PSMA values of prostate lesions were higher in the metastatic group, but significant differences between the metastatic and nonmetastatic groups were only found for PSMA-TV and TL-PSMA values, but not for SUVmax values. The authors of this study concluded that PSMA-TV and TL-PSMA could predict the metastatic risk of PCa. 12 Notably, Kuten et al reported that the presence of metastatic disease was not associated with prostate gland SUVmax value, 16 contrary to our data. The significantly increased SUVmax values in the metastatic group in our study may be due to differences in patient distribution between studies.

Our study has several limitations. The first is its retrospective design, the second is the absence of patients categorized as having low risk according to the D'Amico stratification. The latter can be explained by the fact that baseline ^68^ Ga-PSMA PET/CT imaging is recommended in very few patients that are deemed to have low risk according to clinical features, and thus, causing the absence patients categorized as low-risk based on the D'Amico stratification. On the other hand, although there are many studies in the literature investigating the relationships between ^68^ Ga-PSMA PET/CT and traditional risk systems like the GS and D'Amico, to our knowledge, there are no studies that have investigated its relationship with Candiolo risk classification.

## Conclusion

Our data confirm prior publications that report strong associations between ^68^ Ga-PSMA PET/CT parameters and various clinical risk factors. Quantitative parameters obtained from pretreatment ^68^ Ga-PSMA PET/CT appear to be useful to distinguish high-risk groups (defined by D'Amico and the Candiolo nomogram). More importantly, TL-PSMA appears to be the best parameter as it is the only parameter that can distinguish all risk groups from each other. Baseline ^68^ Ga-PSMA PET/CT parameters could help in the early prediction of the disease outcome and in decision-making for personalized treatment options, thereby improving management and prognosis. Further prospective studies with larger patient populations are required to confirm our results and improve our understanding on this subject.

## References

[1] Siegel R L Miller K D Fuchs H E Jemal A Cancer statistics, 2021 CA Cancer J Clin 2021 71 01 7 33 33433946 10.3322/caac.21654

[2] D'Amico A V Whittington R Malkowicz S B Pretreatment nomogram for prostate-specific antigen recurrence after radical prostatectomy or external-beam radiation therapy for clinically localized prostate cancer J Clin Oncol 1999 17 01 168 172 10458230 10.1200/JCO.1999.17.1.168

[3] EUREKA-2 consortium Gabriele D Jereczek-Fossa B A Krengli M Beyond D'Amico risk classes for predicting recurrence after external beam radiotherapy for prostate cancer: the Candiolo classifier Radiat Oncol 2016 11 23 26911291 10.1186/s13014-016-0599-5 PMC4765202

[4] Vargas H A Grimm J Sala E Hricak H Molecular imaging of prostate cancer: translating molecular biology approaches into the clinical realm Eur Radiol 2015 25 05 1294 1302 25693661 10.1007/s00330-014-3539-5 PMC4994516

[5] proPSMA Study Group Collaborators Hofman M S Lawrentschuk N Francis R J Prostate-specific membrane antigen PET-CT in patients with high-risk prostate cancer before curative-intent surgery or radiotherapy (proPSMA): a prospective, randomised, multicentre study Lancet 2020 395(10231):1208 1216 32209449 10.1016/S0140-6736(20)30314-7

[6] Schmuck S von Klot C A Henkenberens C Initial experience with volumetric 68Ga-PSMA I&T PET/CT for assessment of whole-body tumor burden as a quantitative imaging biomarker in patients with prostate cancer J Nucl Med 2017 58 12 1962 1968 28522740 10.2967/jnumed.117.193581

[7] Grading Committee Epstein J I Egevad L Amin M B Delahunt B Srigley J R Humphrey P A The 2014 International Society of Urological Pathology (ISUP) consensus conference on Gleason grading of prostatic carcinoma: definition of grading patterns and proposal for a new grading system Am J Surg Pathol 2016 40 02 244 252 26492179 10.1097/PAS.0000000000000530

[8] Uprimny C Kroiss A S Decristoforo C ^68^ Ga-PSMA-11 PET/CT in primary staging of prostate cancer: PSA and Gleason score predict the intensity of tracer accumulation in the primary tumour Eur J Nucl Med Mol Imaging 2017 44 06 941 949 28138747 10.1007/s00259-017-3631-6

[9] Koerber S A Utzinger M T Kratochwil C 68 Ga-PSMA-11 PET/CT in newly diagnosed carcinoma of the prostate: correlation of intraprostatic PSMA uptake with several clinical parameters J Nucl Med 2017 58 12 1943 1948 28619734 10.2967/jnumed.117.190314

[10] Onal C Torun N Oymak E Guler O C Reyhan M Yapar A F Retrospective correlation of ^68^ ga-psma uptake with clinical parameters in prostate cancer patients undergoing definitive radiotherapy Ann Nucl Med 2020 34 06 388 396 32221791 10.1007/s12149-020-01462-x

[11] Topuz ÖV Aksu A Erinç S R Tamam MÖ Correlations of ^68^ Ga-PSMA PET/CT in the initial staging of prostate cancer patients Hell J Nucl Med 2021 24 01 60 65 33866340 10.1967/s002449912307

[12] Liu C Liu T Zhang N ^68^ Ga-PSMA-617 PET/CT: a promising new technique for predicting risk stratification and metastatic risk of prostate cancer patients Eur J Nucl Med Mol Imaging 2018 45 11 1852 1861 29717333 10.1007/s00259-018-4037-9

[13] Zschaeck S Andela S B Amthauer H Correlation between quantitative PSMA PET parameters and clinical risk factors in non-metastatic primary prostate cancer patients Front Oncol 2022 12 879089 35530334 10.3389/fonc.2022.879089 PMC9074726

[14] Gabriele D Guarneri A Bartoncini S An external validation of the Candiolo nomogram in a cohort of prostate cancer patients treated by external-beam radiotherapy Radiat Oncol 2021 16 01 85 33952288 10.1186/s13014-021-01814-5 PMC8097839

[15] Utsumi T Suzuki H Ishikawa H External validation of the Candiolo nomogram for high-risk prostate cancer patients treated with carbon ion radiotherapy plus androgen deprivation therapy: a retrospective cohort study Jpn J Clin Oncol 2022 52 08 950 953 35462397 10.1093/jjco/hyac066

[16] Kuten J Mabjeesh N J Lerman H Levine C Barnes S Even-Sapir E Ga-PSMA PET/CT staging of newly diagnosed intermediate- and high-risk prostate cancer Isr Med Assoc J 2019 21 02 100 104 30772960

